# A resistant-starch enriched yogurt: fermentability, sensory characteristics, and a pilot study in children

**DOI:** 10.12688/f1000research.6451.1

**Published:** 2015-06-01

**Authors:** Kayanush Aryana, Frank Greenway, Nikhil Dhurandhar, Richard Tulley, John Finley, Michael Keenan, Roy Martin, Christine Pelkman, Douglas Olson, Jolene Zheng

**Affiliations:** 1 School of Animal Sciences, College of Agriculture, Louisiana State University, Baton Rouge, LA, 70808, USA; 2 Pennington Biomedical Research Center, Louisiana State University, Baton Rouge, LA, 70808, USA; 3 Department of Nutritional Sciences, Texas Tech University, Lubbock, TX, 79409, USA; 4 School of Nutrition and Food Science, College of Agriculture, Louisiana State University, Baton Rouge, LA, 70803, USA; 5 University of California, University of California, Davis, CA, 95616, USA; 6 Ingredion Incorporated, Bridgewater, NJ, 08807, USA

**Keywords:** fatty acids, fiber, home nutrition support

## Abstract

The rising prevalence of obesity and the vulnerability of the pediatric age group have highlighted the critical need for a careful consideration of effective, safe, remedial and preventive dietary interventions.  Amylose starch (RS2) from high-amylose maize (HAM) ferments in the gut and affects body weight.   One hundred and ten children, of 7-8 (n=91) or 13-14 (n=19) years of age scored the sensory qualities of a yogurt supplemented with either HAM-RS2 or an amylopectin starch.  The amylopectin starch yogurt was preferred to the HAM-RS2-enriched yogurt by 7-8 year old panelists ( *P<*0.0001).  Appearance, taste, and sandiness scores given by 13- to 14-year-old panelists were more favorable for the amylopectin starch yogurt than for HAM-RS2-enriched yogurt ( *P<*0.05).  HAM-RS2 supplementation resulted in acceptable (≥6 on a 1-9 scale) sensory and hedonic ratings of the yogurt in 74% of subjects.  Four children consumed a HAM-RS2-enriched yogurt for four weeks to test its fermentability in a clinical trial.  Three adolescents, but not the single pre-pubertal child, had reduced stool pH ( *P*=0.1) and increased stool short-chain fatty acids (SCFAs) ( *P<*0.05) including increased fecal acetate ( *P*=0.02), and butyrate ( *P*=0.089) from resistant starch (RS) fermentation and isobutyrate ( *P*=0.01) from protein fermentation post-treatment suggesting a favorable change to the gut microbiota.  HAM-RS2 was not modified by pasteurization of the yogurt, and may be a palatable way to increase fiber intake and stimulate colonic fermentation in adolescents.  Future studies are planned to determine the concentration of HAM-RS2 that offers the optimal safe and effective strategy to prevent excessive fat gain in children.

## Clinical relevancy statement

Resistant starch (RS) is a type of dietary fiber that people cannot digest, diluting caloric density, but is fermented by bacteria in the intestines into short chain fatty acids that have been shown in other studies to stimulate the production of appetite reducing hormones (see the text). We incorporated resistant starch into a yogurt that was generally accepted by children, increased their dietary fiber consumption and increased colonic fermentation in adolescents. This pilot data suggest the need for a study testing the ability of this yogurt to treat childhood obesity, a vulnerable group where non-food solutions are limited.

## Introduction

The rapidly-growing prevalence of obesity in adults and children requires urgent remedial measures to avert individual and societal health care crises ^1^. Few adult treatment strategies exist ^2^, treating children is more challenging, yet childhood obesity is a growing health concern ^3^. Pediatric vulnerability severely limits the use of pharmacological or surgical interventions. Even dietary treatment with energy and nutrient restriction for weight reduction may be detrimental to growth. Dietary resistant starch (RS) supplementation in food may offer a therapeutic opportunity to attenuate excessive fat gain in infants and children by reducing the caloric density while improving dietary quality ^1, 2^.

RS are dietary carbohydrates that resist cooking processes and enzymatic digestion in the small intestine, are fermented by colonic microbiota and modify the gut flora ^4, 5^. The amount of RS in the human diet has progressively decreased with modern milling and food preparation methods. RS intake in medieval Europe was 50–100g/day ^6^, it is estimated at 30–40g/day ^7^ in developing countries, and has dropped to 3–8g/day in developed countries ^7– 9^. It is unlikely that modern human society will return to a diet of coarsely ground grains and legumes high in RS. However, RS is now available as an ingredient that can be incorporated into breads, cereal products and baked goods that are acceptable to the US population.

Microbiota-derived enzymes are needed to digest complex plant polysaccharides ^10^ and RS-enriched diets increase butyrate-producing Clostridia in rodent feces ^11^. A natural, granular, type 2 RS from high-amylose maize (HAM-RS2) decreases plasma cholesterol and triglycerides, increases satiety, increases insulin sensitivity ^12– 22^, and is anti-adipogenic in adult populations ^23– 30^. HAM-RS2 fermentation in the colon of rodents produces short chain fatty acids (SCFAs) such as acetate, propionate and butyrate that are absorbed through colonocytes, and change colonic microbiota composition ^25, 31, 32^. Butyrate treatment increases gene expression of peptide tyrosine tyrosine (PYY) and proglucagon in ileal, primary colon and cecal epithelial cells of rats; elevates plasma Glucagon-like peptide-1 and -2 (GLP-1, GLP-2), and raises gene expression and protein production of Glucose transporter 2 (GLUT2) ^27, 31, 33– 35^. Clinical studies show that SCFAs increase in response to consumption of HAM-RS2 or RS from potatoes ^36– 38^, that the microbiota of rodents were modified ^39^, and that butyrate was increased in rodents after dietary introduction of human feces ^40^. Since yogurt can deliver dietary fibers to treat constipation in children ^41^, the aim of the current work is to develop a palatable yogurt delivery vehicle for HAM-RS2 that will withstand pasteurization and demonstrate an increase in fecal pH and SCFAs in children and adolescents.

## Methods

### The RS yogurt manufacture

Yogurt mixes were made by incorporating the starches individually into skim milk. The yogurt mixes were pasteurized at 65.5°C for 30 min, cooled to 40°C, inoculated with freshly thawed *Streptococcus thermophilus* (ST-M5) (3.1E+10 cfu/g, 1ml) and *Lactobacillus bulgaricus* (LB-12) (3E+10 cfu/g, 1ml) (Chr. Hansen Inc., Milwaukee, WI) per 3.785L (1 gallon), then incubated at 40°C until they reached a pH 4.5, and held at 4°C overnight. Blueberry puree (20% w/w) was incorporated into the yogurt the following day and amylopectin starch (15 g, control, AMIOCA ^®^ corn starch, Ingredion Incorporated, Bridgewater, NJ) or HAM-RS2 (15g, HI-MAIZE ^®^ 260 resistant starch, Ingredion Incorporated, Bridgewater, NJ) per 237ml serving was added to the yogurt (Creamery, College of Agriculture, LSU). A high performance liquid chromatography (HPLC) peak was detected in our HAM-RS2 sample and RS accounted for 38.2% of the sample.

### *In vitro* testing

HAM-RS2 30g/237ml yogurt was used for *in vitro* testing. Six samples were prepared, coded, and tested blindly with half subjected to pasteurization. A modified Englyst method was used to quantify glucose release ^4^. Intact granular structure of the starch was evaluated using birefringence light microscopy.

### Sensory study

The Institutional Review Board (IRB) granted an exemption #HE13-1 (January 16, 2013) from continued oversight for the sensory study conducted in two groups of children evaluating the two yogurts. Ratings of satisfaction with the appearance, color, aroma, taste, thickness, sandiness, and palatability of each type of yogurt were scored by 110 children without communication. Ninety-one children were 7–8 years old (younger) and 19 were 13–14 years old (elder). The younger children were more willing to volunteer for the sensory study than the elder.

Subjects with no dairy or starch-related allergies were recruited from The Louisiana State University Laboratory School and parental consent to participate was obtained along with the children’s assent. Participants were given yogurt samples in 85g cups with a snap-on lid. Cups were coded with a random three-digit number. Disposable plastic spoons and napkins were provided to prevent contamination between samples. Prior to the sensory evaluation, the children were provided with a “warm-up” yogurt sample to avoid the “first sample effect” due to possible previous consumption of other food items, and a cup of drinking water was provided to rinse their palate between samplings. Two evaluation forms were used, one with a face scale for the younger panelists and the other with a preference rating form for the older panelists, and clearly explained to each age group. The younger panelists indicated their yogurt preference by circling “smiling face (☺) as yes”, scored as 3, “neutral face (😐) as neither like nor dislike”, scored as 2, or “sad face (☹) as no”, scored as 1. The elder panelists evaluated the yogurt on a 1–9 scale (1-dislike extremely, 2-dislike very much, 3-dislike moderately, 4-dislike slightly, 5-neither like nor dislike, 6-like slightly, 7-like moderately, 8-like very much, 9-like extremely) for appearance, color, aroma, taste, thickness, and sandiness. The elder panelists evaluated the yogurt thickness by checking 1-too thin, 2-just about right, or 3-too thick; and the sandiness as 1-not grainy, 2-just about right, or 3-too grainy. Elder panelists answered the question “Is this product acceptable?” with 2-“yes” or 1-“no” answer.

### Clinical study

The four-week pilot clinical trial was approved by the PBRC Institutional Review Board (IRB28012) and registered ( http://clinicaltrials.gov/, NCT01338571) to determine the effects of consuming HAM-RS2-enriched yogurt on fecal pH and fecal SCFAs, pre- and post-consumption, in a healthy child and three healthy adolescents. The subjects (a 6-year-old female, two 10-year-old African-American females, and a 14-year-old Caucasian male) were recruited through the PBRC recruiting department. Parents signed a consent form and subjects signed an assent form. Subjects with gastrointestinal disease, on medications with the potential to alter the intestinal bacterial microbiota such as antibiotics and subjects with allergies to corn were specifically excluded.

Subjects were weighed in the morning on an electronic scale in light street clothing without shoes or outer clothing and with pockets emptied. The electronic scale (Model 450, GSE Inc., Livonia, MI, USA) was calibrated daily using standardized weights and quarterly by an external service. Parents were given stool-collecting kits and instructed to collect a stool specimen from their child for 3 consecutive days at baseline and after 4 weeks of yogurt consumption. They were provided with ice packs, coolers and were instructed to return stool samples in the coolers to the research site on the day they were collected so they could be stored at -70°C until analysis.

Children were given HAM-RS2 10g plus 1g per year of age daily ^42^ which was 16, 20, or 24g for the four subjects. A fresh supply of yogurt was given to the parents weekly and the daily yogurt was divided into servings at breakfast and dinner.

Measurements of fecal SCFAs and pH were previously published elsewhere ^18^. Briefly, the frozen fecal specimens were thawed, homogenized and further diluted to wet sample in distilled water (0.5g/5ml). The pH was measured using a combination electrode. Samples were then acidified with metaphosphoric acid (250g/L, 1ml) containing ethyl-butyric acid (2g/L) as an internal standard. The mixture was vortexed, and centrifuged at 4°C for 10 minutes at 8,000 rpm to remove solids in the homogenized samples and syringe-filtered (33mm, Millipore, Billerica, MA). The filtrate was put into a gas chromatograph (GC) auto-sampler vial and capped. SCFAs in the effluent were analyzed using gas-liquid chromatography. The GC conditions (115°C for 0.1 min) were increased to 150°C for 0.1 min in increments of 10°C, then to 170°C for 2 min at increments of 11°C. The injector temperature was 250°C. Helium was the carrier gas with a flow rate of 60 ml/min and splitless injection was 60 ml/min. Single SCFAs were determined by retention time based on standards and the relative concentrations calculated based on the ratio of the peak areas of the sample to the internal standard.

### Statistical analysis

The RS content differences of yogurt samples prepared with or without pasteurization were determined using the Student *t*-test (SAS 9.3, SAS Institute Inc., Cary, NC). The sensory data obtained from children were analyzed with a Randomized Block Design using panelists as blocks (GLM, SAS 9.1), and the paired *t*-test for HAM-RS2 score minus amylopectin score was performed. Differences between the types of yogurt were determined by differences of least squares mean ± SEM. The clinical data analysis of feeding yogurt to the four subjects was performed with the Student *t*-test (weight) and paired *t*-test (change in weight) (SAS 9.1). Alpha was set at 0.05.

### Ethics

All procedures followed were in accordance with the ethical standards of the responsible committee on human experimentation (institutional and national) and with the Helsinki Declaration of 1975, as revised in 2000.

## Results

### Resistant starch in yogurt

**Figure 1.**
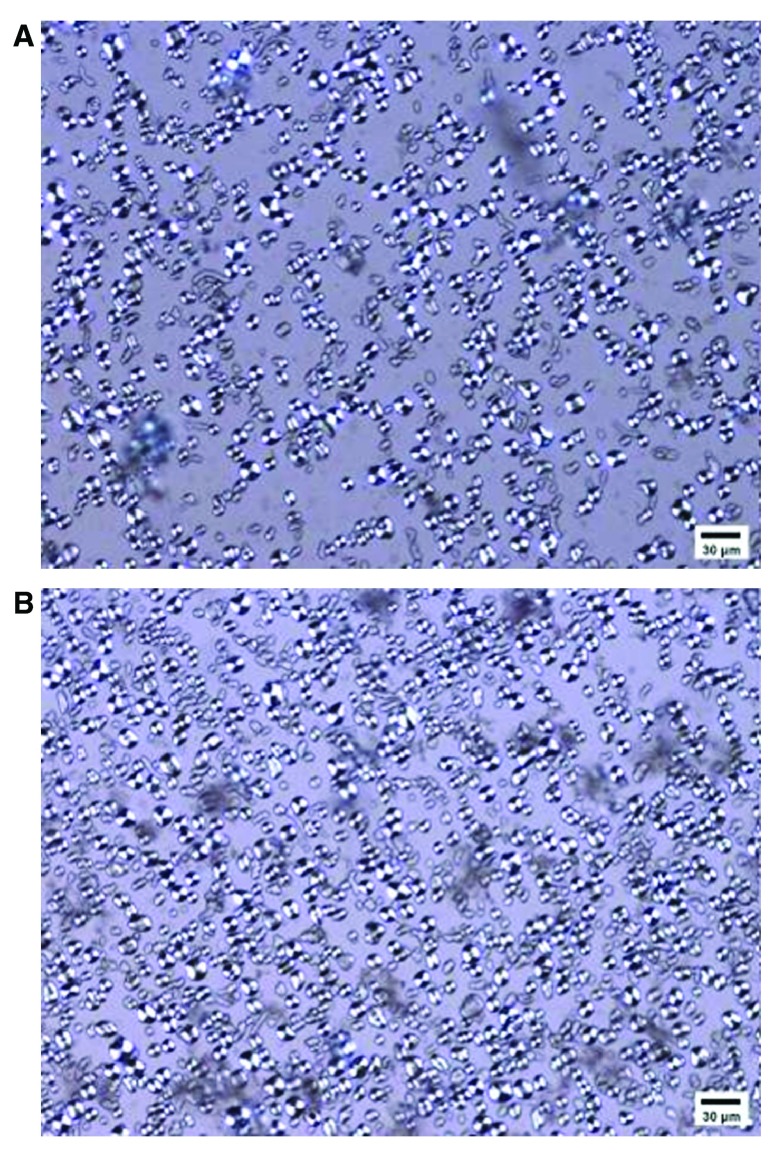
HAM-RS2-enriched yogurt observed using polarized light microscopy (200x). Bar=30μm.

The glucose release was detected by a modified Englyst method. A light microscope (200x, Leitz Wetzlar, Ortholux II, Ernst Leitz GmbH, Wetzlar, Germany) revealed morphologies of the starch granules in the yogurts as having an equal presence of birefringence indicating an intact granular structure ( Figure 1). The RS content of the six yogurt samples varied minimally (from 45% to 51% on a dry weight basis with values of 51%, 45%, or 48% for the unpasteurized samples, and 45%, 45%, or 46% for the pasteurized samples ( *P*>0.05).

### Sensory results for HAM-RS2 versus amylopectin starch

***Group 1: Ninety-one 7- and 8-year-old panelists.*** The average scores were 1.538 for the HAM-RS2-yogurt and 2.143 for the amylopectin starch yogurt. The difference was -0.604±0.1 (t=6.05, *P<*0.0001) which indicated that the amylopectin starch yogurt was preferred over the HAM-RS2-yogurt.

***Group 2: Nineteen 13- and 14-year-old panelists.*** No score differences were detected in color (6.95±0.36 vs. 7.05±0.28) and aroma (7.84±0.24 vs. 7.47±0.32) for the amylopectin starch compared to the HAM-RS2-yogurt, respectively ( Table 1, *P*>0.05). However, appearance (6.84±0.34 vs. 4.58±0.38), taste (6.95±0.32 vs. 4.84±0.49), thickness (6.74±0.48 vs. 4.47±0.37), and sandiness (6.26±0.37 vs. 3.05±0.36) scores for the amylopectin starch yogurt were higher than for the HAM-RS2-yogurt ( *P<*0.005).

**Table 1.** Comparison of the appearance, color, aroma, taste, thickness, and sandiness score (on a 1 to 9 scale in which 1 was the least desirable ranking and 9 was the most desirable ranking) of the HAM-RS2 (treatment) versus the amylopectin starch (control) yogurt when evaluated by 13- and 14-year-old children.

Type of starch present in yogurt	Appearance	Color	Aroma	Taste	Thickness	Sandiness
Amylopectin starch	6.84 ^a^ ± 0.34	6.95 ^a^ ± 0.36	7.84 ^a^ ± 0.24	6.95 ^a^ ± 0.32	6.74 ^a^ ± 0.48	6.26 ^a^ ± 0.37
HAM-RS2	4.58 ^b^ ± 0.38	7.05 ^a^ ± 0.28	7.47 ^a^ ± 0.32	4.84 ^b^ ± 0.49	4.47 ^b^ ± 0.37	3.05 ^b^ ± 0.36

^ab^Means without a common superscript are significantly ( *P*<0.05) different from each other.

On a 1–3 scale (1-too thin, 2-just about right, or 3-too thick), the amylopectin starch yogurt (2.26±0.13) was judged slightly thicker than just about right while the HAM-RS2-yogurt (1.16±0.09) was judged as too thin ( *P<*0.0001, Table 2).

**Table 2.** Comparison of the thickness score (1 = too thin, 2 = just about right, or 3 = too thick), the sandiness score (1 = not grainy, 2 = just about right, or 3 = too grainy), and the acceptability score (1 = no (not acceptable) or 2 = yes (acceptable)) of the HAM-RS2 (treatment) versus the amylopectin starch (control) yogurt when evaluated by 13- to 14-year-old children

Type of starch present in yogurt	Thickness Score 1 = too thin 2 = just about right 3 = too thick	Sandiness Score 1 = not grainy 2 = just about right 3 = too grainy	Acceptability Score 1 = no (not acceptable) 2 = yes (acceptable)
Amylopectin starch	2.26 ^a^ ± 0.13	1.95 ^b^ ± 0.12	2.00 ^a^
HAM-RS2	1.16 ^b^ ± 0.09	2.84 ^a^ ± 0.12	1.74 ^b^

^ab^Means without a common superscript are significantly ( *P*<0.05) different from each other.

Using a 1–3 scale (1-not grainy, 2-just about right, and 3-too grainy), the HAM-RS2-yogurt (2.84±0.12) was judged as too grainy but was acceptable to 74% of the children of 13–14 years of age, while the amylopectin starch yogurt (1.95±0.12) was judged as just about right ( Table 2, *P<*0.0001).

The amylopectin starch yogurt was always judged as acceptable ( Table 2) and its acceptability on a 1–2 scale (1-not acceptable or 2-acceptable) was significantly higher than for the HAM-RS2-yogurt ( *P<*0.05). The sensory study indicated that children preferred the amylopectin starch yogurt more than the HAM-RS2 added yogurt.

Sensory raw data from study participants Participants were asked to rate amylopectin starch- and HAM-RS2-containing yogurts for preference (7–8 years old; 1–3 scale, 1 being dislike, 3 being like) or appearance, color, aroma, taste, thickness, and sandiness (13–14 year olds; 1–9 scale, 1 being dislike extremely, 9 being like extremely) ^45^. Click here for additional data file. Copyright: © 2015 Aryana K et al. 2015 Data associated with the article are available under the terms of the Creative Commons Zero "No rights reserved" data waiver (CC0 1.0 Public domain dedication).

### Clinical study

All adolescent participants finished the HAM-RS2-yogurt and returned the empty containers during the weekly clinic visits with no complaints regarding taste or compliance issues related to consumption of the yogurt.

One 10-year-old had a BMI of 19.8 kg/m ^2^ and the other had a BMI of 27.1 kg/m ^2^. The 14-year-old had a BMI of 31.5 kg/m ^2^. All were otherwise healthy. The pre-pubertal child gained 1.9kg (39.5 to 41.4 kg). One of the adolescent females gained 3.2kg (49.6 to 52.8kg), the other one gained 0.4kg (69.5 to 69.9kg) and the adolescent male gained 1.7kg (89.9 to 91.6 kg) ( *P*>0.05).

**Figure 2.**
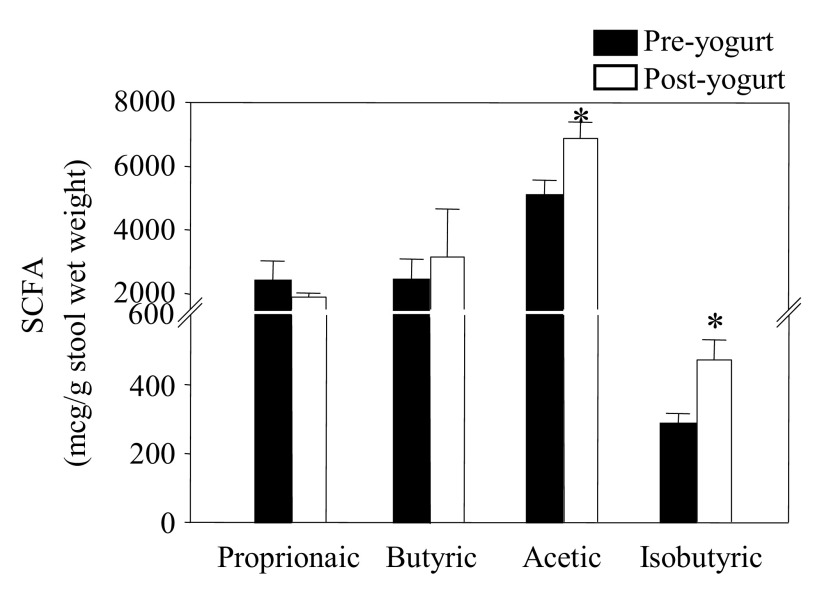
Stool SCFAs increased ( *P*<0.05) in adolescents post-yogurt treatment. The pre-pubertal child was not included.

**Figure 3.**
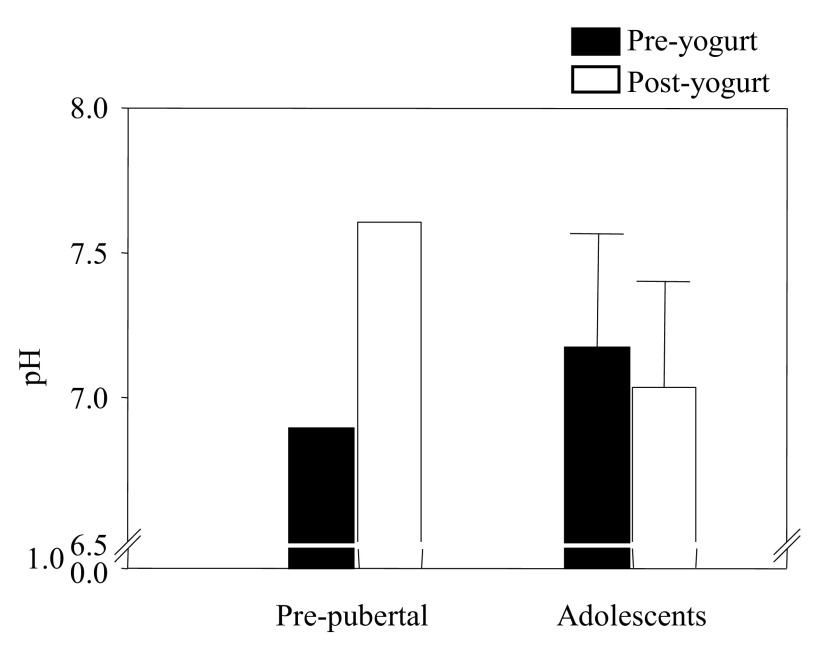
Stool pH was reduced ( *P*=0.1) in adolescents post-yogurt treatment. The pH was increased in the pre-pubertal child.

SCFAs (μg/g wet stool weight) from carbohydrate fermentation were increased in the adolescent participants; in ascending order, butyrate (23%, 2,410±691 to 3,144±1,509µg, *P=*0.09), acetate (26%, 5,078±492 to 6,870±515µg, *P=*0.02), but not propionate (2,387±645 to 1,889±120µg, *P*>0.05). The isobutyrate from protein fermentation increased (39%, 285±31 to 471±58μg, *P=*0.01) ( Figure 2). The stool pH of the adolescents was mildly reduced at the end of the fourth week with a trend toward a lower pH (2.8%, from 7.2±0.4 to 7.0±0.35, *P=*0.1, Figure 3).

The pre-pubertal participant responded to HAM-RS2-enriched-yogurt differently than the three adolescent children with an increase in stool pH (from 6.89 to 7.62). The stool SCFAs were decreased; in ascending order, isobutyrate (35%, from 526 to 186µg), butyrate (39%, from 4,028 to 1,571µg), acetate (52%, from 8,328 to 4,336µg), and propionate (65%, from 2,870 to 1,877µg) over the 4-week study.

Raw data for clinical study Four participants (a 6-year-old female, two 10-year-old African-American females, and a 14-year-old Caucasian male) were weighed and their weights recorded. Parents were given stool-collecting kits and instructed to collect a stool specimen from their child for 3 consecutive days at baseline and after 4 weeks of yogurt consumption. They were provided with ice packs, coolers and were instructed to return stool samples in the coolers to the research site on the day they were collected so they could be stored at -70°C until analysis. Children were given HAM-RS2 10g plus 1g per year of age daily which was 16, 20, or 24g for the four subjects. SCFA content of samples was analysed by GL-chromatography and determined by retention time based on standards and the relative concentrations calculated based on the ratio of the peak areas of the sample to the internal standard ^46^. Click here for additional data file. Copyright: © 2015 Aryana K et al. 2015 Data associated with the article are available under the terms of the Creative Commons Zero "No rights reserved" data waiver (CC0 1.0 Public domain dedication).

## Discussion

The prevalence of childhood obesity has increased 2- to 3-fold in just the last 25 years globally (see review 2). Childhood obesity is associated with co-morbidities similar to adults including hypertension, dysglycemia, dyslipidemia, inflammation and endothelial dysfunction ^2^. Supplementing other treatment approaches with behavioral interventions may increase long term participation and is felt to be more important in the pediatric population than for adults ^2^, but meta-analyses show prevention or treatment strategies to be ineffective. Currently, no truly effective pharmacological options are available for weight management, and surgery is restricted to a highly selected subgroup of very obese adolescent individuals. Medication and surgery have safety concerns in growing children and their efficacy is uncertain in the pediatric age group ^43^. Novel treatments for childhood obesity offering safety, efficacy and acceptability are urgently needed ^44^. Desirable attributes of an intervention for pediatric obesity include a preventive measure that attenuates excess fat accumulation while allowing for normal growth. RS is a natural food ingredient with a low risk profile that attenuates body fat accretion in experimental animal models, and is an excellent candidate to effectively combat childhood obesity. This feasibility study suggests that HAM-RS2-enriched foods likely alter microbiota composition, and this is supported by the increase in fecal SCFA content and lower pH. Yogurt was a generally acceptable vehicle for providing HAM-RS2. The yogurt cultures fermented lactose (milk sugar) and the RS granules in the final yogurt product were not damaged.

Enriching the diet with RS which has been refined out of the US diet will improve dietary quality and may help to ease the severity of pediatric obesity. Although the amylopectin starch yogurt was preferred, our studies confirmed the general acceptability of incorporating HAM-RS2 into yogurt through taste and sensory testing in 91 7- to 8-year-olds and 19 13- to 14-year-old volunteers. The four subjects in our pilot study that consumed the HAM-RS2-enriched yogurt twice a day for weeks established the feasibility of feeding the HAM-RS2-enriched yogurt to children. We demonstrated a trend toward a reduction of pH and documented a significant increase in the SCFA content of the stools of the adolescent children. This agrees with previous studies that have found that adding HAM-RS2 to rodent diets reduced abdominal fat in association with increased fermentation ^20, 21, 37, 44^. Supplementing the diet with RS will need to be acceptable and palatable or children are likely to reject it in favor of low-fiber alternatives. Overall, the HAM-RS2-yogurt in the taste testing was acceptable to 74% of the children in the 13- to 14-year-old group, but 24% less acceptable in younger children. The knowledge that RS is healthy may increase the adoption of RS fortified foods, such as yogurt.

People eat for volume and consume fewer calories when food has a lower caloric density ^38^. RS and other dietary fibers reduce the caloric density of food ^12, 13, 44^. RS is present in many different sources, which offers the opportunity to choose the RS with the greatest success in reducing or controlling body weight ^17^. We have previously shown that the HAM-RS2 supplementation produces a 30% reduction in intestinal fat deposition in wild type *C. elegans* ^28^ and in rodents the same also reduced body fat ^25, 44^. Longer-term controlled studies are needed to determine if the reduced adiposity seen in animal models will occur in human populations. In a human pilot study, a HAM-RS2 (15g/day) supplemented diet enhanced insulin sensitivity by 56.5% in men over two to three months, which suggests that lower amounts of HAM-RS2 may also be efficacious. Beneficial changes in adiposity may occur over longer treatment periods ^12, 13, 21, 22^, and lower amounts of RS may further improve palatability – an important factor for long-term consumption.

Children maintain weight loss better than adults ^11^. Although it is not clear why the pre-pubertal child in this study did not respond in the same way as the adolescents, it could represent differences in her intestinal microbiota or her pre-treatment diet which was not controlled nor queried. Further research will be necessary to explore the differential role of diet and the intestinal microbiota on the fermentation of RS before puberty. Weight gain in all of the children during the 4-week study may reflect the fact that they were growing.

## Conclusion

The current study showed the acceptability and feasibility of using yogurt to deliver RS to adolescents which caused a change in SCFA and probably changed the gut microbiota. These preliminary data suggest the need to evaluate differences that may exist in the microbiota before and after puberty to determine whether the non-response of the pre-pubertal child represented an outlier or a real effect in pre-pubertal children. These preliminary results will need confirmation in a controlled trial so that the effects of growth can be taken into account in evaluating weight changes in longer-term studies using yogurt as a vehicle to deliver the functional food component HAM-RS2 in a range of doses into everyday foods that consumers enjoy. Our data encourage controlled studies in children and adolescents testing insulin sensitivity, effects on body weight, and potential differences between pre-pubertal and adolescent children in their microbiota response to RS. Hopefully, increased consumption of reduced-calorie foods in combination with increased physical activity ^12^ will reduce weight gain, help to maintain a healthier weight, and lead to future improvements in public health for adults and adolescents alike.

## Data availability

The data referenced by this article are under copyright with the following copyright statement: Copyright: © 2015 Aryana K et al.

Data associated with the article are available under the terms of the Creative Commons Zero "No rights reserved" data waiver (CC0 1.0 Public domain dedication). 

*F1000Research*: Dataset 1. Sensory raw data from study participants, 10.5256/f1000research.6451.d47918 ^45^

*F1000Research*: Dataset 2. Raw data for clinical study, 10.5256/f1000research.6451.d48004 ^46^

## Consent

Informed consent was obtained from all patients being included in the study.
