# Supplementary material for: A resistant-starch enriched yogurt: fermentability, sensory characteristics, and a pilot study in children
Source: F1000Res. 2015 Jun 1;4:139. [Version 1] doi: 10.12688/f1000research.6451.1 (PMC4712773; doi:10.12688/f1000research.6451.1)
Supplement: Peer review history

# Peer review: Referee response for version 1

**DOI:** 10.5256/f1000research.6923.r10019

**Published:** 2015-08-26

**Authors:** Joanne Lupton

**Affiliations:** 1 Department of Nutrition and Food Science, Texas A&M University, College Station, TX, USA

The authors are addressing an important issue -- the development of foods for children that will not impede their growth, but also not contribute to adiposity, that children will like to eat, and that will withstand pasteurization.  In their case they have chosen to add a specific resistant starch (HAM-RS2)- to yogurt. There was a good rationale for them to select this resistant starch as previous studies done in rats, and some done in humans, have shown beneficial results. They performed a series of sensory tests comparing the yogurt with resistant starch to their control yogurt (containing amylopectin). Except for the color of the product all other sensory attributes were rated better for the control than the yogurt containing resistant starch.The statistical analyses appear to be acceptable except there was no stated allowance for multiple T tests.  Despite this, it is clear that the children liked the control better than the one containing resistant starch.  Instead of calling this product a "bust" the authors have chosen to call this a success as a certain percentage still liked the experimental product sufficiently to eat it. One wonders how the abstract would have read if the resistant starch containing product scores were reversed with the controls. One would imagine that they would have been delighted if not ecstatic.

The "clinical trial" in children was important to do, as it provided information as to the acceptability of the resistant starch over time.  However, there were only 4 children in this trial and one, who was pre-pubescent (age 6), was different from the others (2) 10 y and (1) 14, and the results from that individual were also different from the other 3. In one way this is really a preliminary test of the resistant starch, not a trial, as there was no control.  All 4 children ate the resistant-starch yogurt, in amounts based, in part, on their ages. Again, the results of this trial were disappointing if weight gain was a primary outcome measure.  All gained weight, and for some this was substantial.  The resistant starch appeared to be fermented to SCFA, but the values for pH and SCFA were reported in a previous paper.  If weight gain, or gain of muscle mass and not fat stores was an important endpoint and these children were still growing, the study would have benefitted by a control group of the amylopectin containing yogurt. 

The title of the paper seems incomplete as it doesn't mention the aim of the pilot study.  I've tried to come up with the purpose of the clinical trial, but the fact that I'm unsure what the goal was is impeding me.  Is it testing the fermentability of the resistant starch product?  If so, it should provide pH and SCFA data.  Is it testing whether or not children will eat this product for a month, then it should say that.  If it's about weight gain/loss then it needed a control. 

In summary, I think this idea of accepting a paper and then asking experts in the field to comment is excellent, and I'm glad to be part of the process.  These authors are experts in what they are doing, and they have found some important information about a natural, granular, type 2RS from high-amylose maize (HAM-RS2).  Others working in this field can benefit from the fact that they presented their data in what could be characterized as a preliminary study.  However, there are two points that bother me about the interpretation of their data.  First, they put a very positive spin on the HAM-RS2 intervention, when by almost all accounts this was not a positive outcome.  This really should be toned down.  Second, the authors make statements about the benefits of fermentability that come across as "facts" when rather this is an open and unresolved issue.  For example, when discussing HAM-RS2 they say "decreases plasma cholesterol and triglycerides, increases satiety, increases insulin sensitivity and is anti-adipogenic in adult populations."  Although they show several citations that have shown this, they have not reviewed the entire literature, and they make the statement as if it is fact.  This needs to be qualified.   In a different place they say "People eat for volume and consume fewer calories when food has a lower caloric density."   This is Barbara Rolls's hypothesis, but not everyone would agree with this statement.  In fact, Rolls herself says it mostly applies to men, not women.  So, please go back through your comments that are stated as FACTS and perhaps modify them.

I have read this submission. I believe that I have an appropriate level of expertise to confirm that it is of an acceptable scientific standard.

# Peer review: Referee response for version 1

**DOI:** 10.5256/f1000research.6923.r8858

**Published:** 2015-06-30

**Authors:** Patrick O’Neil

**Affiliations:** 1 Department of Psychiatry and Behavioral Sciences, Medical University of South Carolina, Charleston, SC, USA

There is much current attention to the role of the gut, especially gut microbiota, in the regulation of body weight. At the same time the US and other countries face escalating rates of childhood and adolescent obesity, suggesting the importance of obesity prevention efforts, of which there have been few shown to have significant impacts. This study examines one innovative potential approach to preventing obesity targeting food components, specifically the incorporation of dietary resistant starch into a common food item, yogurt. The study focused on the acceptability of the sensory characteristics of yogurt enhanced with one such resistant starch, compared to yogurt enhanced with a control amylopectin starch, among pre-adolescent and adolescent subjects. A small pilot 4-week trial also looked at the effects of the resistant starch when consumed by 4 subjects.

The sensory evaluation study was well-designed with tight controls and a sizable N. Assessment of various sensory attributes was rather comprehensive among the adolescent sample, which unfortunately was substantially smaller than the sample of 7- to 8-year olds who could not be expected to make the finer sensory distinctions on which the adolescents were queried. The actual resistant starch content of the various yogurt samples was verified by analyses.

The sensory evaluation study showed that the subjects consistently preferred the control yogurt to that containing the resistant starch.  The adolescents found the resistant starch yogurts to be inferior to the control yogurts on appearance, taste, thickness, sandiness and acceptability. About 25% of the adolescent subjects rated the resistant starch as unacceptable. Among the younger children, whose only ratings were a smiling face, neutral face or sad/frowning face, the resistant starch yogurt was more strongly disliked, with nearly two-thirds of subjects awarding it a frowning face. The discussion does not adequately address these results, which show consistently that the sensory characteristics of the resistant starch yogurt preparation are not well received by children and adolescents. The conclusion that “…our studies confirmed the general acceptability of incorporating HAM-RS2 into yogurt…” seems to fly in the face of these results.

The very small pilot clinical trial provided some support for the hypothesis that the resistant starch would produce changes in gut microbiota, findings worth following up with a larger study. It also showed that despite the limited acceptability ratings, the products could be consumed over a 4-week period. However, the weight gain seen among all four subjects is disturbing. Although the discussion dismisses this finding by saying that it may reflect growth, the amounts of weight gain seen over 4 weeks were 0.4, 1.7, 1.9 and 3.2 kg.  Certainly the latter three gains, if continued over a year, would be quite excessive. Given that the point of this dietary intervention is to prevent excessive weight gain, these preliminary findings are cause for considerable concern.

The authors have conducted a well-designed study of the feasibility of this innovative dietary intervention which has the purpose of obesity prevention. The findings show that it is possible to get children and adolescents to consume yogurt containing resistant starches, but more development is necessary to produce a food product with adequate acceptability.  Aside from that, however, the assumption that this product, if consumed regularly, might avert excess weight gain needs further study, as the limited results here suggest its effect may be just the opposite of that which was expected and desired.

I have read this submission. I believe that I have an appropriate level of expertise to confirm that it is of an acceptable scientific standard.
